# The study of ableism in population health: a critical review

**DOI:** 10.3389/fpubh.2024.1383150

**Published:** 2024-04-17

**Authors:** Kara M. Mannor, Belinda L. Needham

**Affiliations:** Center for Social Epidemiology and Population Health, Department of Epidemiology, University of Michigan School of Public Health, Ann Arbor, MI, United States

**Keywords:** ableism, disability, health equity, population health, critical review

## Abstract

Over the past three decades, health equity has become a guiding framework for documenting, explaining, and informing the promotion of population health. With these developments, scholars have widened public health’s aperture, bringing systems of oppression sharply into focus. Additionally, some researchers in disability and health have advocated for utilizing socially grounded frameworks to investigate the health of disabled people. Yet, naming ableism, much less operationalizing it for the empirical study of health, remains scant. This paper critically reviews the study of ableism as a social determinant of disabled people’s health within population health research. First, we provide an orientation to the present state of this literature by looking to the past. We briefly trace a history of traditional approaches to studying disability and health and alternatives that have emerged from critiques of the individualized lens that has dominated this work. Next, we delineate the operation of ableism across social levels. We characterize how ableism has been studied in population health in terms of levels of analysis (intrapersonal, interpersonal, institutional, and structural) and measures of interest. To conclude, we discuss hinderances to and promising avenues toward population health research that advances health equity for disabled people.

## 1 Background

The disparate death-making of the COVID-19 pandemic (1, 2) brought to mainstream public health consciousness the devastating impact of disability-based discrimination in public health preparedness schemes, clinical decision making, institutional care arrangements, and other domains. This consciousness raising can be indexed by the spike in essays published in public health journals that highlight barriers, stigma, and social norms that adversely impact access to healthcare and constrain the life chances of disabled people (3–6). Alongside these commentaries, researchers have made further calls for public health as a field to explicitly recognize disabled people as a group whose marginalization warrants attention in eliminating health disparities and realizing equitable health outcomes (7, 8). In times of crisis, there are often more opportunities to push social issues that have long fomented outside of public awareness to the spotlight where they are not as easily ignored. Perhaps bodies piled outside nursing homes and efforts to expose unjust and discriminatory pandemic critical care practices (9, 10) have opened more avenues in public health to discuss disabled people’s experiences of systemic social disadvantage, but these ideas are certainly not that new.

Scholars have been engaged in discussions around reorienting public health and medicine’s relationship to disabled people for decades (11). Nearly 15 years ago, Drum and colleagues heralded an “unprecedented paradigmatic shift” (12) in the study of disability and public health. And 5 years before the pandemic’s onset, Krahn and colleagues (13) outlined their case that disabled people meet the field’s accepted definition of a “health disparity population,” couched in a broad sketch of the historical disadvantages that disabled people have suffered. All this talk about change in public health research is undoubtedly due to the popularization of the social model of disability. As we discuss further, this social model seeks to shift the understanding of disability from the individual level to barriers in the broader environment (14, 15). However, as we demonstrate, public health scholars have yet to seriously take up a key domain for study that the social model of disability names as its focal point: the oppression of disabled people.

Critical reviews conceptually analyze and synthesize a body of literature towards the development of new questions or hypotheses rather than making a claim about quality or rigor (16). The task of suggesting new directions in population health approaches to the study of disability requires taking stock of the current literature *and* understanding how the field got where it is today. Ideas about disability that are available directly to researchers inform the questions they (neglect to) ask about the state of disabled people’s health. In population health^1^ research, it is taken as self-evident that disability is a natural or medical phenomenon rather than a social category with dynamic meanings and expressions that are shaped by political, economic, and cultural contexts (18–20). These unspoken assumptions coalesce in research aims, findings, and proposals for future investigations, restricting opportunities to study the social origins of disabled peoples’ health. Therefore, we situate this critical review in a brief historical account of traditional and alternative approaches to studying disability and health.

We describe ableism as a social determinant of health as it operates across four levels of social analysis: intrapersonal, interpersonal, institutional, and structural. Next, we describe the extent to which population health researchers have studied factors that map onto our ableism levels in relationship to the health outcomes of disabled people. Our findings include key patterns and themes in the literature, through which we make legible the assumptions and logics that hinder rather than advance this research. In synthesizing these findings, we suggest future areas of inquiry and opportunities for engagement with theoretical work across disciplines to guide practice and action that advances health equity for disabled people.

### 1.1 Traditional and alternative approaches to studying disabled populations

Disability is a complex and evolving concept. Human bodies and minds have always varied, but the significance of that variation – from the unremarkable to a stigmatized status – cannot be understood outside of historical and social context. The disability studies literature is replete with models of disability with differing levels of differentiation, nuance, and foci (21, 22). Here, we briefly outline key examples of these models to illustrate overarching conceptual differences that are relevant to the study of ableism.

In the US, concepts of disability are entwined with the reorganization of labor and social relationships following the emergence of capitalism and industrialization. Indeed, capitalist logics of individualism, independence, and productivity (23) converge with the ascendancy of clinical judgment as authoritative validating device (20) to inform what is known as the medical model of disability (12).

The medical model of disability has been the dominant way of explaining disability in professional fields and tends to pervade “common-sense” thinking about disability (14). According to this model, most problems that disabled people encounter are the direct result of their individual impairments. The research and practice corollaries of the medical model include an emphasis on interventions aimed at “restoring” disabled people to (some approximation of) “normality” or the need for disabled people to become appropriately adjusted to their impairments (24).

This thinking is embedded across prevention schemes within traditional public health research and practice (12). If the normal body cannot be preserved through the primary prevention of disability, then the public health imperative becomes lessening the “weight” of disabled individuals and their impairments on the general population. This is perhaps no more starkly illustrated than in the use of disability adjusted life years (DALYs) to quantify population health “burdens” in public health research and surveillance (25). In DALY calculus, disabled people never live full years. By definition, their lives are made fractional by disability weights (26). The medical model is therefore operationalized in public health via DALYs, making disability synonymous with problems of bodily function or structure.

Though dominant, the medical model of disability is not the only model of disability used within public health. The social model of disability, which was first described as such by Mike Oliver, emphasizes the role of the physical and social environment in the production of disability (14). Oliver’s articulation was built on work by the Union of Physically Impaired Against Segregation (UPIAS), a political organization of persons with physical impairments in Britain working towards the replacement of institutions for physically impaired people with alternative arrangements that provided for their full participation in society (27). In their model, UPIAS distinguished between two concepts: impairment as deficient bodily structure or function and disability as a construct imposed on top of people’s impairments through the systematic exclusion of people with impairments from daily and civic life (27).

This explicitly political approach sparked a paradigm shift that has ignited people’s imaginations towards new possibilities for disabled people for a half century. Fixing our attention on the social has opened avenues for building disability-centered political movements, the passage of national legislation with significant public health impacts, and developing a positive collective identity for disabled people (24). In a public health context, the social model is often identified as integrated into the WHO’s International Classification of Functioning, Disability and Health (ICF), which discards previous definitions of disability as individual limitation and proposes disability as “an umbrella term for impairments, activity limitations or participation restrictions” (28).

While the ICF’s incorporation of aspects of the social model of disability addresses some important limitations of the medical model, the ICF is not without controversy (some of which stems from limitations of the social model). Briefly, the main critiques from disability studies scholars include: a false universalization of the experiences of disabled people across other social positions (29, 30); sublimation of impairment as an object of analysis through the impairment/disability binary which is not adequate for understanding embodied experiences like pain and fatigue (31); and defining disability *as oppression* rather than *involving oppression*, which forecloses opportunities to study social and health outcomes in relation to identity formation, group belonging, or other positive aspects of disability (24).

This cursory overview of disability models illustrates that the lens that researchers use to study disability significantly influences the phenomena they are able to explore. A medical model allows inquiries into problems of isolated bodies and minds. To speak of or analyze ableism becomes ontologically irrelevant when this model is operant. This is not so much a neutral scientific pursuit (which is implied when disability is cast as a *de facto* marginalized status), but a politically convenient one insofar as it “leaves the social world unchallenged” and the goals of the medical or rehabilitation expert unquestioned (14).

### 1.2 Ableism as a system of oppression

Disability studies scholarship has advanced the understanding of systemic oppressive processes that cast disability as a “diminished state of being human” (32). Because it is the term most frequently used in the US, we use the term ableism^2^ to denote these oppressive processes (34). Like other systems of oppression, ableism operates across multiple levels.

*Internalized ableism* includes the ways in which disabled people accept negative stereotypes endorsed by the dominant culture and the relatively low status of disabled people as a group in society (35). Internalized ableism can manifest as disavowing disabled identity and minimizing impairments or attempting to “pass” – where passing is not just hiding one’s impairments, but mitigating the discomfort of others through humor, charm, or other strategies (36). Because ableist logic tends to individualize disability, disabled people tend to have fewer resources and opportunities to develop cultures that explicitly reject negative stereotypes of disability (36).

*Personally-mediated or interpersonal ableism* is disability-based discrimination that occurs through day-to-day interactions between individuals and includes overt, intentional acts of prejudice and more subtle, covert experiences of indignity (37). Overt forms of interpersonal ableism can be hostile, including the use of shaming language, the avoidance of disabled people in public, and even violence perpetrated by caregivers or other individuals. Such overt acts also manifest as false benevolence, including expressions of pity and unsolicited praise (38). More subtle experiences of discrimination in everyday interactions between individuals are also called microaggressions. These microaggressions manifest as denial of life experiences (e.g., expressing disbelief that a disabled person is employed or is partnered), public demands that infringe upon privacy (e.g., strangers asking about a person’s diagnosis), assumptions of helplessness (e.g., insisting on assisting a disabled person who does not need or want support doing some activity), and other acts (37).

Oppression also operates at the institutional level through “practices and policies within institutions that result in the systematic denial of resources and opportunities to members of subordinate[d] groups….[and is] maintained by the laws, organizational guidelines, or traditions of an institution” (39). While the Americans with Disabilities Act (ADA) and other legislation prohibits discrimination against disabled people across domains of public life in the US, research has demonstrated that anti-discrimination legislation often fails to address broader historical and cultural factors that perpetuate inequity for marginalized groups (40). Examples of *institutional ableism* include systems within schools that segregate disabled students from their peers and educational opportunities (41); pervasive assumptions in medical settings about disabled peoples’ inability to communicate about their health; assumptions about the relationships between health, disability, and quality of life that compromise quality of healthcare (42); lack of knowledge among physicians about accommodations and lack of accessible equipment in the clinic (43); and workplace practices that disproportionately decline interviewing qualified disabled applicants, and refuse accommodations (44).

Drawing on Iris Marion Young’s concepts of structural injustice and social-structural processes, disability can be understood as a social-structural position. That is, disabled persons “differ from persons differently situated in the range of options available to them and in the nature of the constraints on their action” (45). The social positioning of disabled people (and the attendant health impacts) cannot be understood as a function of individual actions or a single policy, but through “many policies, both public and private, and the actions of thousands of individuals acting according to normal rules and accepted practices [that] contribute to producing these circumstances” (45). Further, social structural processes do not only constrain certain groups, but they enable others to act. One way that *structural ableism* marks the physical landscape of the US is through features of built environments. Scholars have linked architectural design for a default “universal white, male, nondisabled body” back to forces – such as eugenic ideologies – that shape public spaces as “a site of management, surveillance, and control” (46). Additional forms of structural ableism include forms of confinement that disproportionately impact disabled people and policies and practices that constrain asset accrual and other pathways to economic security (47). We next characterize how ableism has been studied as a social determinant of health in terms of levels of analysis and measures of interest.

## 2 Methods

We used OVID to search the Medline database for English-language articles published from January 2010 to July 2023. Article eligibility criteria included: (1) quantitative or qualitative analysis; (2) primary aim(s) characterized some aspect of ableism (according to the levels of ableism described above) in relation to a health or healthcare outcome of a disabled group in the US; and (3) disabled groups defined in terms of impairment, functional limitation, identity/social position, or a specific health condition framed in terms of disability or a disabling process. While there are connections between ageism and ableism given the association between aging and disability, they are not fully overlapping constructs (48). Therefore, this review does not include studies that exclusively studied ageism or self-concepts of aging in relation to health without distinguishing disability-based discrimination. The full search strategy and more details of our methods are presented in the Supplemental material.

Using the definitions provided above, we mapped measures and themes from eligible studies, to characterize levels of ableism studied in population health. In the mapping process, we utilized text from the methods and background sections of the articles that described and conceptualized the measures and themes. We also extracted data on disability definitions and measures used and theories and frameworks that informed these studies.

## 3 Findings

### 3.1 Overall characteristics

After the removal of duplicates, the search yielded 1,617 articles, 41 of which were determined to be eligible for this review and are characterized in Table 1. The majority used quantitative methods (58.5%; *n* = 24) and among those articles, 66.7% (*n* = 16) had an analytic primary aim that assessed the relationship between some form of ableism and a health outcome among a disabled group. Most articles collected data on disability status via self-report (58.5%, *n* = 24). However, markers of disability status were highly heterogenous and included specific diagnoses, impairments, functional limitations, ability to work, and social identity. Notably, 26.8% (*n* = 11) of articles did not collect data directly from a disabled group. These articles solicited information from clinicians (*n* = 8), focused on the experiences of parents with disabled children (*n* = 2), or utilized area-level data on disability attitudes of the general public (*n* = 1). Just under half of the articles explicitly used a theory or conceptual framework to guide their research (48.8%, *n* = 20). Nine articles (22%) used the term ‘ableism,’ although mostly as a passing mention rather than operationalizing a system of oppression for analysis (49–57).

**Table 1:** Levels of ableism studied in the population health sciences literature, January 2010 – July 2023.

Study	Study type	Level of ableism	Type of ableism measure or qualitative theme	Health outcome	Disabled population of interest	Disability definition	Disability measurement	Theories or frameworks
Agaronnik et al. (58)	Qualitative	Institutional	*Clinician attitudes or practices*: e.g., patients with disabilities are challenging	Healthcare access or quality	General	Historically marginalized identity group; ADA definition of disability	Not a study population (interviewed clinicians)	Social model of disability
Agaronnik et al. (59)	Qualitative	Institutional	*Clinician attitudes or practices*: e.g., misattributing cancer symptoms to underlying disabilities *Clinical setting*: e.g., inaccessible medical diagnostic equipment	Healthcare access or quality (cancer screening, diagnosis)	Persons with mobility disabilities	Requires use of assistive device and/or ADL assistance; ICF definition of disability	Self-report of functional limitation	ICF
Ames et al. (60)	Qualitative	Institutional	*Clinician attitudes or practices*: e.g., inappropriate interaction with patient *Clinical setting*: e.g., lack of accommodations	Healthcare access or quality	Children with physical, cognitive, communication, and/or social functioning disabilities	Medical complexity involving ≥2 organ systems and technology dependency, functional impairment or high health care use	Parent-report (interviewed parents)	Author’s conceptual framework of interpersonal disability-based discrimination in health care
Byrappagari et al. (61)	Quantitative, descriptive	Institutional	*Clinician attitudes or practices*: survey on barriers and willingness to treat patients with developmental disability	Dental caries and other oral health outcomes	Persons w/ developmental disabilities	Conditions due to an impairment in physical, learning, language, or behavior areas that impact everyday life	Not a study population (surveyed clinicians)	None
Carolan et al. (50)	Qualitative	Institutional	*Workplace discrimination*: e.g., bias in hiring process	Health trajectory	Women with chronic health conditions	Chronic health condition or impaired mobility	Self-report of health condition	Cumulative inequality theory; intersectionality
Cordova et al. (82)	Qualitative	Interpersonal Institutional Structural	*Everyday discrimination*: e.g., shunned by family, perceived disability stigma in community *Clinician attitudes or practices*: e.g., being put down by service providers *Built environment*: e.g., inaccessible public transit and community settings	Substance use, mental health	Latinos with physical disabilities	Physical impairment limiting ability to perform normal and daily activities for ≥3 months	Self-report of impairment	None
Friedman and VanPuymbrouck (57)	Quantitative, analytic	Structural	*Societal attitudes*: framed in relation to US histories of institutionalization and measured via aggregate DA-IAT scores at the state level	State-level Medicaid expenditure (framed as quality of life)	General	Historically marginalized group	Not a study population (data from general public)	Structural ableism
Friedman (51)	Quantitative, analytic	Structural	*Societal attitudes*: framed in relation to structures of disadvantage and measured via aggregate DA-IAT scores at MSA level	Quality of life (Personal Outcome Measures® scale)	BIPOC w/ intellectual or developmental disabilities	Historically marginalized group	Not defined	None
Goreczny et al. (62)	Quantitative, descriptive	Institutional	*Clinician attitude or practices*: study-specific attitude/belief survey	Healthcare access or quality; quality of life	Persons with intellectual or developmental disabilities	Not defined	Not a study population (surveyed service providers)	None
Harrison et al. (78)	Qualitative	Institutional Structural	*Clinician attitudes or practices*: e.g., assumptions about cognitive ability or ability to perform self care *Clinical setting*: e.g., denial of accommodations to access health information *Societal attitudes*: e.g., negative societal views about capacities of persons with VI (as linked to access to accommodations)	Health literacy	Women with visual impairments	Impairment	Self-reported legally blind despite correction (low or no vision)	IOM’s Health Literacy Framework
Hawkins et al. (83)	Quantitative, analytic	Interpersonal Institutional Structural	Environmental barriers to community participation measured by CHIEF-SF *Everyday discrimination*: e.g., problem with people’s attitudes towards you at home or in the community *Policies and practices*: e.g., barriers created by government policies and programs *Built environment:* e.g., difficulty with design and layout of buildings in community	Community integration/participation	Veterans with single or multiple physical, psychological, and/or emotional injuries	Limitations and restrictions in the activity and participation domains of the ICF	Self-reported injury	ICF; social cognitive theory
Hughes et al. (63)	Qualitative	Institutional	*Clinician attitudes or practices*: e.g., lack of disability-related education and information among providers *Clinical setting*: e.g., lack of accessible exam tables	Health literacy	Women with physical disabilities	Impairment	Self-reported mobility impairment with a duration of at least 1 year	None
Iezzoni et al. (64)	Qualitative	Institutional	*Clinical setting:* e.g., lack of accessible mammography and radiation therapy equipment	Healthcare access or quality (cancer screening, diagnosis)	Women with early-stage breast cancer and chronic difficulty walking or use of wheeled mobility aids	ICF definition of disability	Self-reported disability or mobility aid use	ICF
Iezzoni et al. (65)	Qualitative	Institutional	*Clinician attitude or practices:* knowledge of responsibilities under ADA, whether practice welcomes disabled patients	Healthcare access or quality	General	ADA definition of disability	Not a study population (interviewed clinicians)	None
Jones and Miller (66)	Quantitative, descriptive	Institutional	*Clinician attitude or practices:* perception of disabled persons as similar to or different and inferior to oneself as measured by ATDP	Healthcare access or quality (oral health)	General	Not defined	Not a study population (surveyed dental students)	None
Khazem et al. (74)	Quantitative, analytic	Internalized Interpersonal	*Self-stigma:* Perceived burdensomeness *Perceived stigma*: felt stigma from Jacoby Stigma Scale	Mental health (suicide attempt or ideation)	Persons with physical disabilities	ADA definition of physical disabilities	Self-reported physical disability (included blindness/low vision, mobility difficulties)	Interpersonal theory of suicide
Khazem et al. (52)	Quantitative, analytic	Internalized Interpersonal	*Self-stigma:* Perceived burdensomeness and thwarted belonging from INQ *Perceived stigma*: felt stigma from Jacoby Stigma Scale	Mental health (suicide attempt or ideation)	Persons with disabilities impacting their vision or mobility	Not described	Self-reported vision (blindness/low vision, etc.) or mobility (double amputation, paraplegia, etc.) disability	Interpersonal theory of suicide
Lagu et al. (49)	Qualitative	Institutional	*Clinician attitude or practices:* e.g., disabled people are entitled, burdensome *Clinical setting:* building inaccessibility, lack of accessible equipment	Healthcare access or quality	General	Not described	Not a study population (interviewed clinicians)	None
Leding-ham et al. (53)	Qualitative	Institutional Structural	*Clinician attitude or practices:* e.g., being left out of treatment session activities due to disability *Clinical setting:* e.g., lack of accommodations in treatment settings *Built environment:* lack of accessible transportation to treatment	Healthcare access or quality (substance use treatment)	Adults with disabilities who misuse opioids (dually disabled)	Not described	Self-reported disability	Critical disability theory; intersectional stigma
Magasi et al. (67)	Qualitative	Institutional	*Clinician attitude or practices:* e.g., general disrespect for disabled people *Clinical setting:* e.g., lack of accessible exam equipment	Healthcare access or quality (cancer screening, diagnosis)	Women with physical disabilities	Not described	Self-reported physical disability	CBPR aligned with tenets of the disability rights movement
Magasi et al. (54)	Qualitative	Internalized	*Self-stigma*: e.g., reject disability label based on negative perceptions of disabled people	Quality of life; healthcare access or quality	Cancer survivors with disabilities	ADA definition	Self-reported disability; ACS six disability questions	ICF; social constructivism
Mitra et al. (68)	Qualitative	Institutional	*Clinician attitude or practices:* e.g., viewed as incapable of bearing children *Clinical setting:* e.g., lack of accessible exam equipment	Pregnancy outcomes (low birth weight, premature birth)	Women with physical disabilities who have been pregnant	Not described	Self-reported physical disability or health condition affecting ability to walk or arms/hands	None
Monden et al. (75)	Quantitative, analytic	Internalized Interpersonal	SCI QOL Stigma-SF *Self-stigma*: e.g., I felt embarrassed about my injury *Perceived stigma:* e.g., because of my injury, people avoided looking at me	Depression, quality of life, participation, perceived disability	Persons with SCI	Not described	Record of SCI in injury surveillance system	Systems approach
Morris et al. (69)	Quantitative, analytic	Institutional	*Clinician attitude or practices*: physical exam completion; surveyed patients using Patient Perceptions of Quality of Care subscales	Healthcare access or quality	General	Not described	Self-reported gross and fine motor, visual, hearing, speech and language, learning, or cognitive disabilities	None
Namkung and Carr (87)	Quantitative, descriptive	Interpersonal Institutional	(88) EDS *Everyday discrimination*: lack of respect, blemish of character, insulted/harassed subscales *Workplace discrimination:* not hired for a job, etc. *Service discrimination:* denied a bank loan, etc.	Mental health	Adults with physical disability across lifecourse stages	Functional limitation	Self-reported some limitation in any item of SF-36 from MIDUS	Stigma theories; lifecourse theory
Namkung and Carr (89)	Quantitative, analytic	Interpersonal	*Everyday discrimination*: lack of respect, blemish of character, insulted/harassed from Williams et al. (1997) EDS subscale	Mental health (depressive symptoms; positive/negative affect)	Adults with physical disability across lifecourse stages	Condition that impairs ability to perform ADLs or IADLs	Self-reported some limitation in any item of SF-36 from MIDUS	Stigma theories; stress process; lifecourse theory
Nandam et al. (70)	Quantitative, analytic	Institutional	Accommodation needs and availability at screening *Clinician attitude or practices*: e.g., explanation of exam *Clinical setting*: e.g., accessible mammogram machine	Healthcare access or quality (mammography prevalence and screening guideline compliance)	Women with cerebral palsy	Chronic condition present from birth with motor-related impairments (weakness, balance deficits, spasticity, dystonia, ataxia)	Not described	None
Remillard et al. (85)	Mixed method	Structural	Challenges with transportation *Societal attitudes*: e.g., wheelchair users should not use public transit conveyed through remarks, eyerolls, etc. *Built environment:* e.g., lack of accessible parking spaces, presence of steps on buses	Community integration/participation	Persons aging (60–79 years old) with mobility disability	Acquired functional limitation in early- to mid-life	Self-reported serious difficulty walking or climbing stairs	None
Rimmer et al. (71)	Quantitative, descriptive	Institutional	*Clinician attitude or practices*: e.g., presence of care team protocols for and comfort with discussing burn patients’ sexual intimacy	Quality of life	Burn injury survivors	Physical limitations and disfigurement associated with burns	Not a study population (interviewed burn care professionals)	None
Rogers et al. (72)	Quantitative, analytic	Institutional	*Clinician attitude or practices*: ‘received poorer treatment than other people from doctors or hospitals’ item from Williams et al. (1997) EDS	Health trajectory	Older adults (over 50 years old) with disability	ADL difficulty or dependence	Self-reported ADL difficulty or dependence in ambulating, bathing, dressing, eating, toileting, or transferring.	None
Sánchez et al. (80)	Quantitative, analytic	Internalized Structural	*Self-stigma*: e.g., ‘I estrange myself from others because I am a mental health consumer’ from SSS-S *Societal stigma:* extent to which someone believes most people will devalue a person with mental health problems from PDD	Quality of life	Persons with severe mental illness	Mental illness and difficulty completing ADLs and/or IADLs	Self-reported primary diagnosis of severe mental illness	ICF
Schniedewind et al. (73)	Quantitative, descriptive	Institutional	*Clinician attitude or practices*: denial of or failure to provide qualified interpreter for healthcare visit	Preventable adverse health event	Deaf ASL user	Distinct cultural group	Self-identified as Deaf ASL user to Idaho Council on the Deaf and Hard of Hearing	None
Seng et al. (77)	Quantitative, analytic	Internalized Interpersonal	Stigma Scale for Chronic Illnesses 8-item version *Self-stigma*: e.g., I felt embarrassed about my illness *Perceived stigma:* e.g., Because of my illness, people were unkind to me	Mental health (depression, anxiety, catastrophizing)	Adults with migraine-related disability	Missed or reduced activity levels; role restriction, role prevention, poor emotional function	Migraine Disability Assessment score; Migraine-Specific Quality of Life Questionnaire score	Social ecological model of migraine; stigma theories
Shakarchi et al. (90)	Quantitative, descriptive	Interpersonal	*Everyday discrimination*: 5 items from Williams et al. (1997) EDS in HRS	Physical and mental health outcomes	Persons with sensory impairment	Visual and/or hearing impairment	Self-reported vision and hearing ability	None
VanPuymbrouck et al. (55)	Quantitative, descriptive	Institutional	*Clinician attitude or practices*: implicit attitudes as measured by as DA-IAT and explicit preference for disabled people by health providers	Healthcare access or quality; chronic health conditions	General	Not described	Not a study population (surveyed clinicians)	None
Venkatesan et al. (86)	Quantitative, analytic	Interpersonal Institutional	*Everyday discrimination*: 9 items from Williams et al. (1997) EDS *Major experiences of discrimination*: e.g., unfairly denied housing or a bank loan	Behavioral health outcomes	Persons aging (at least 50 years old) with TBI	TBI	Diagnosis on medical record	None
Wang et al. (56)	Quantitative, analytic	Internalized Interpersonal	Stigma Scale for Chronic Illnesses 8-item version *Self-stigma*: e.g., I felt embarrassed about my illness *Perceived stigma:* e.g., Because of my illness, people were unkind to me	Mental health (depression, anxiety)	General	Not defined	Self-identified disability	None
Whittle et al. (79)	Qualitative	Internalized Interpersonal Structural	*Self-stigma*: e.g., feeling shame around receiving disability services *Perceived stigma*: e.g., being avoided or feeling judged as lazy due to disability *Experiences of obstructive bureaucracy*: e.g., policies and practices related to government aid that cast recipients as potential malingerers	Health trajectories	Persons with type 2 diabetes and/or HIV enrolled in a food assistance program	Work limiting condition; stigmatized condition	Physician-certified diagnosis	Structural stigma; context of neoliberal welfare reform
Wong et al. (84)	Quantitative, analytic	Interpersonal Institutional Structural	Environmental barriers to community participation measured by CHIEF-SF and HACE *Everyday discrimination*: e.g., problem with people’s attitudes towards you at home or in the community *Policies and practices*: e.g., barriers created by government policies and programs *Built environment:* e.g., difficulty with design and layout of buildings in community	Community integration/participation	Persons with neurologic disorders	Acquired neurological disorder	Physician diagnosis of TBI, SCI, or stroke	ICF; authors’ transactional model of participation
Young et al. (76)	Quantitative, analytic	Internalized Interpersonal	*Self-stigma*: e.g., sense of shame or anxiety about condition via Stigma Scale for Chronic Illness-I *Perceived stigma*: e.g., lost friends due to condition via Stigma Scale for Chronic Illness-E	Mental health	Persons with migraine or epilepsy	Ability to work	Ability to work score; Migraine Disability Assessment score (for migraine patients only)	Stigma theories
Zuckerman et al. (81)	Qualitative	Interpersonal Institutional Structural	*Perceived stigma*: e.g., community members think children with disabilities are improperly disciplined *Clinician attitudes or practices:* burdens of multiple appointments and negative interactions at clinic create barriers to follow-up *Machismo*: traditional view of Latino male gender roles creates barriers, particularly among fathers, for accepting disability and ASD diagnosis	Healthcare access or quality (diagnostic delays)	Latino children (age 2–10) with ASD	Not described	Parent report of child’s diagnosis (interviewed parents)	None

ACS, American community survey; ADL, activities of daily living; ASD, autism spectrum disorder; ASL, American sign language; ATDP, attitude toward disabled persons - form O; BIPOC, black, indigenous and people of color; CBPR, community-based participatory research; CHIEF-SF, Craig Hospital inventory of environmental factors short form; DA-IAT, disability attitudes implicit association test; EDS, everyday discrimination scale; HACE, home and community environment survey; HRS, health and retirement study; IADL, instrumental activities of daily living; ICF, international classification of functioning, disability and health; IOM, institute of medicine; INQ, interpersonal needs questionnaire; MIDUS, midlife development in the U.S survey; MSA, metropolitan statistical area; PPD, perceived devaluation-discrimination scale; QOL, quality of life; SCI, spinal cord injury; SSS-S, self-stigma scale short form; TBI, traumatic brain injury; VI, visual impairment.

### 3.2 Studying ableism as a social determinant of health

Institutional ableism was the most common level of ableism studied alone or in addition to other levels (69.5%; *n* = 27). The levels of ableism studied across articles are depicted in Table 2. Most articles focused their inquiry on one level of ableism (61.0%; *n* = 25). We did not categorize any articles in this set as having measures or themes that reflected all four levels of ableism.

**Table 2:** Levels of ableism studied in the population health sciences literature, January 2010 – July 2023.

Internalized	Interpersonal	Institutional	Structural	*N* (%)
		**✓**		19 (46.3%)
**✓**	**✓**			5 (12.2%)
	**✓**	**✓**	**✓**	4 (9.8%)
			**✓**	3 (7.3%)
	**✓**			2 (4.9%)
	**✓**	**✓**		2 (4.9%)
		**✓**	**✓**	2 (4.9%)
**✓**	**✓**		**✓**	2 (4.9%)
**✓**				1 (2.4%)
**✓**			**✓**	1 (2.4%)

Almost half of the articles focused on the institutional level alone in investigating ableism in relation to health of a disabled group (46.3%; *n* = 19) (49, 50, 55, 58–73). The clinic was the primary domain of interest. All but one article on workplace discrimination studied clinician attitudes or practices related to disabled patients or accessibility features of clinical settings. A substantial proportion (42.1%; *n* = 8) of this subset are articles that collected data exclusively from health providers (49, 55, 58, 61, 62, 65, 66, 71). Authors of these articles often named the persistence of discrimination in healthcare despite federal statutes that mandate equality in access to care as a motivating factor for their studies. Thus, clinician attitudes and practices emerge as a key area of interest. Most of these articles discussed the need for training to improve interactions with disabled patients and to improve knowledge related to legal requirements for accommodations, with some proposing cultural competency frameworks and/or healthcare partnerships with disability community groups (31, 54, 55, 58, 59, 62) as potential avenues for better training. Three articles discussed the role of limitations on Medicaid reimbursements as possibly contributing to negative dynamics in clinical settings for disabled patients (61, 68, 73). Only five articles in this subset explicitly utilized a theory or conceptual framework. Two used the ICF (59, 64) and one used the social model of disability (58) to point their investigations toward social and environmental factors. One article used grounded theory principles to create a conceptual model of disability discrimination in healthcare (60). The article on workplace discrimination in relation to the health trajectories of women with chronic health conditions was informed by cumulative inequity theory and intersectionality (50).

The next most common pattern was studying internalized and interpersonal ableism together (12.2%; *n* = 5) (52, 56, 74–76). These articles were interested in the relationship between forms of self-stigma, perceived stigma, and mental health outcomes among disabled groups. Two articles used the interpersonal theory of suicide to guide their studies, while a third drew on the broader literature of stigma theories (52, 74). Seng and colleagues also interested in the role that stigma plays in health, however, they more explicitly conceptualized stigma as operating across intrapersonal, interpersonal, and structural levels (77). They proposed a social-ecological model in which stigma arises when migraines prevent individuals from fulfilling a society’s normative behavioral expectations. As such, authors acknowledged that while pain reduction and therapeutic coping strategies are likely important in mitigating migraine stigma, they also highlighted the need for exploring broader societal dynamics, such as norms within workplace and school settings. Only one article in this review was interested in internalized ableism alone in the form of self-stigma (54).

Eleven additional articles focused on structural ableism, either alone (7.3%; *n* = 3) or in addition to other levels (19.5%; *n* = 8). Six of these articles were interested in attitudes, norms, or discriminatory experiences, but were classified as having structural ableism measures or themes in this review because of the explicit conceptualizations employed by authors (51, 57, 78–81). For example, Whittle and colleagues used a qualitative approach to explore how changes in welfare benefits policies have impacted ‘the lived experience of disability and stigma’ for individuals living with type 2 diabetes or HIV (79). While participants in this study reported discriminatory encounters at benefits offices or through other social services systems, the authors used theory to situate these experiences within the broader context of neoliberalization of the welfare state regime in the US. In addition, Friedman and VanPuymbrouck used measures of disability-related implicit bias at the state level to assess the relationship between ‘disability prejudice’ and state expenditures for home and community-based services to maximize community living for disabled people (57). The authors couched their investigation in a narrative of shifting policy and social-cultural changes that have both banished disabled people behind the walls of institutions and created opportunities for them to live and be seen as members of their communities. This historical and conceptual grounding allows the authors to present the health-related consequences of ableism beyond a function of individual psychology or operation at the interpersonal level to a structural relationship, consistent with Young’s definition.

Five additional articles with themes or measures at the structural level examined the physical built environment, including transit design, building architecture, and land development. Three of these articles were interested in community participation as defined by the ICF (82–84). Two articles that used the ICF as a guiding framework (beyond definitions), also studied everyday discrimination at the interpersonal level and policies and practices at the institutional level (53, 85), consistent with ICF’s account of interacting domains.

The final four articles of this review studied interpersonal ableism or included both interpersonal and institutional ableism measures (86–90). Three of the articles were interested in mental health outcomes. At the interpersonal level, articles used measures of everyday discrimination (related to disability status). The two articles that added measures at the institutional level studied major instances of discrimination in the workplace (87) or across multiple institutional domains (86).

## 4 Discussion

This review demonstrates that the study of ableism and the health of disabled populations is still an emerging topic area for population health. This result is most evidently indexed by the lack of the use of ableism as a concept to understand the myriad ways that disabled people are impacted by systems of oppression. In using levels of ableism to characterize the literature, we found that ableism is most often studied at the institutional level – specifically in clinical settings and focused on the attitudes and practices of healthcare providers. We discuss the implications of these and other findings as they relate to hinderances and promising avenues for future population health research on ableism.

This critical review identified 41 studies in the population health literature on ableism and the health outcomes of disabled populations published across a 13.5-year period. That volume of literature is far out-paced by population health studies focusing on other systems of oppression – such as racism (91). This is not to say that the quantity of literature is the most important marker of a field’s development or contribution to public health knowledge. However, studies of population health and racism, for example, are not only more numerous but public health scholars have contributed to more robust conceptual work to operationalize how racism impacts health at the group level. Notably, just over half of the articles used any theory or framework to guide their analysis. In fact, authors of most studies in this review did not use the term ableism at all, much less harness it as a framing concept for their analyses. Instead, they employed more general concepts like discrimination and stigma applied to disability as marginalized status, only sometimes explicitly informed by theory. This lack of conceptual engagement leaves population health research at rote conclusions regarding the health impacts of ‘negative attitudes and beliefs’ about disability. In the best-case scenario this does little to unsettle ableism as the status quo and in the worst case presents ableism as an individual and ‘natural’ phenomenon, artificially narrowing possibilities for intervention. This also forecloses opportunities to advance scholarship towards asking more critical questions about how and why attitudes and beliefs persist or become entrenched within broader structural processes, or the ways in which ableism impacts health beyond the attitudinal sphere.

This review also found that a disproportionate volume of population health research on ableism is focused on healthcare. This is significant in the context of a global pandemic that has disproportionately claimed the lives of disabled people. As noted in our results, important work has pushed for more and better disability-related training through a cultural competency lens to address ableism among health trainees and practitioners (92).

The preponderance of studies interested in institutional ableism within healthcare settings only – especially those that did not collect data directly from disabled individuals – also points to some thorny questions about this body of literature. That is, to what extent do population health researchers understand disabled people to be reliable narrators of their experiences in clinical settings? And, to what extent are population health researchers imagining disabled people beyond the patient role in formulating their research questions? These epistemological issues point to several possible interventions within population health to improve research on disabled populations. We again underscore the importance of theory-informed inquiry. To surface and disrupt these more insidious ways that ableism informs the research process, training and research institutions must commit to policies and practices that deepen engagement with critical scholarship that challenges traditional and often unarticulated assumptions about disability (93, 94). The value of this engagement is illustrated by articles in this review, like Whittle and colleagues (79), whose theoretical grounding allowed the authors to discuss issues of competency training of social services providers as one path to stigma prevention and address how stigma gets perpetuated and reproduced through the structural operation of benefits provision. It also allowed the authors to proffer more radical (meaning *from the root*) avenues of research that would inform efforts to destigmatize the lived experience of being disabled – such as universal basic income programs.

Our findings also suggest a role for a structural competency approach to training across health-related disciplines and practices (95). Originally conceived to train medical students to better understand and address how structural inequities show up in the clinic, structural competency has been reformulated into a set of guidelines and practices for epidemiologists to introduce epistemic humility into the research process and address paradigmatic challenges of a ‘structural turn’ within the field (96). Although they do not directly address issues specific to disability and ableism, some social epidemiologists have been advocating for alternatives to positivist epistemological frameworks in the field (97) to provide a foundation for using qualitative methodologies that can inform deeper and richer explanations of social and health phenomena. The qualitative studies that we highlight in this review demonstrate this explanatory potential. Further, such paradigmatic shifts open creative possibilities for disrupting and transforming knowledge production dynamics that (re)produce intersecting logics of systems of oppression (98) including racism, settler colonialism, heterosexism, ableism, and more.

Training alone is insufficient to developing population health research that can meaningfully inform action on health inequities related to ableism. Our introduction outlines some transformative impacts that disabled people have had on the direction of disability research across a wide range of fields. Yet, representation of disabled people among principal investigators in the health sciences has remained low (99). Our findings on the operation of ableism in clinical settings and epistemological issues within population health speak to the consequences of excluding disabled people from this field as producers of knowledge. Much work is needed to remedy these issues, including supporting opportunities that allow disabled researchers to lead research programs informed by critical scholarship. Additionally, researchers (irrespective of their relationship to disability) should continue to adopt and adapt participatory methods that allow disabled people to meaningfully contribute to health research (100). These practices align with and are supported by the broader methodological developments suggested above.

## 5 Limitations

One limitation of this scoping review is the data source. We used the Medline database to characterize the study of ableism and disabled peoples’ health within population health sciences research. This review did not cover research journals that are only indexed in social services-focused databases, and therefore may have underestimated the volume of research. Secondly, this review is limited by the terms used to capture studies of ableism in the literature. Population health literature uses a wide range of terms to describe ableism, and as this review found, does not utilize a key term like ableism that specifically names the forms of marginalization that disabled people experience. Therefore, this review may not have captured articles that exclusively use more conceptually ambiguous terms (such as ‘barriers’) to study ableism and disability health.

## 6 Conclusion

The impact of ableism in population health is an important area of investigation, however, atheoretical inquiries risk reproducing harmful ableist norms rather than illuminating pathways toward their elimination. Engagement with theory and other frameworks is a critical step if population health researchers are to produce evidence informing public health action that is consistent with calls for health equity. Beyond theory, our review underscores the need and potential for deeper disciplinary changes toward more critical knowledge production around ableism as a social determinant of health. As this body of literature continues to grow, future reviews should seek to better understand the extent to which population health researchers are using theories of intersectionality to study ableism in relationship to additional systems of oppression.

## Data availability statement

The original contributions presented in the study are included in the article/Supplementary material, further inquiries can be directed to the corresponding author.

## Author contributions

KM: Conceptualization, Data curation, Formal analysis, Writing – original draft, Writing – review & editing. BN: Conceptualization, Methodology, Supervision, Writing – original draft, Writing – review & editing.

## Footnotes

^1^This paper utilizes the terms “population health” and “public health” interchangeably, following the guidance of Diez Roux (17).

^2^Some scholarship distinguishes ableism from disablism, where ableism promotes the unattainable ideal of the “species-typical individual citizen” (32) and disablism is the systematic exclusion and marginalization of disabled people from society (33).
