# Supplementary material for: The study of ableism in population health: a critical review
Source: Front Public Health. 2024 Apr 17;12:1383150. doi: 10.3389/fpubh.2024.1383150 (PMC11061527; doi:10.3389/fpubh.2024.1383150)
Supplement: Supplementary file 1 [file Data_Sheet_1.docx]

**Appendix A. Critical review methods details**

We used OVID to search the Medline database for English-language articles published from January 2010 to July 2023. We employed keyword and adjacency searches (detailed in the below search strategy) to capture three main concepts related to the aim of this review: 1) disability; 2) measures of ableism; and 3) health or healthcare access-related outcomes. Following the removal of duplicates, the search yielded 1617 articles. In the screening process, we primarily assessed article titles and abstracts and utilized full texts as needed for clarity. Article eligibility criteria included: 1) quantitative or qualitative analysis; 2) primary aim(s) characterized some aspect of ableism (according to the levels of ableism described in the manuscript) in relation to a health or healthcare outcome of a disabled group in the US; and 3) disabled groups defined in terms of impairment, functional limitation, identity/social position, or a specific health condition framed in terms of disability or a disabling process. While there are connections between ageism and ableism given the association between aging and disability, they are not fully overlapping constructs (1). Therefore, this review does not include studies that exclusively studied ageism or self-concepts of aging in relation to health without distinguishing disability-based discrimination.

Using the definitions provided in the manuscript, we mapped measures and themes from eligible studies, to characterize how ableism is studied in population health. In the mapping process, we utilized text from the methods and background sections of the articles that described and conceptualized the measures and themes. We mapped articles according to the level the authors assigned to the theme or measure, with one exception (described below). In instances where the authors did not characterize their measures or themes in terms of a level of ableism, we used their descriptions (e.g., self-stigma) to assign the level. Ames and colleagues characterized their conceptual model of healthcare discrimination as ‘interpersonal’ given that interactions took place between two or more people (2). We concede that discrimination within healthcare settings often manifests as interactions between two or more individuals. However, these interactions are intertwined with sets of formal and informal institutional policies and practices and can have consequences involving access to resources that exceed the interpersonal level. For this reason, we characterized the ableism measure in the study by Ames and colleagues (2) as institutional rather than interpersonal.

**OVID search strategy**

1. disabled persons/
2. disab*.ab,ti,kw.
3. ((mobility or functional or physical or mental or hearing or vision or visual or self-care) adj2 (limit* or impair* or difficult*)).ab,ti,kw.
4. (stigma* or attitude* or oppress* or discrimination or ableism).ab,ti,kw.
5. 1 or 2 or 3
6. 4 and 5
7. exp Cardiovascular Diseases/
8. exp Neoplasms/
9. exp Self-Injurious Behavior/
10. exp Diabetes Mellitus/
11. exp Mood Disorders/
12. exp Substance-Related Disorders/
13. Body Mass Index/ or exp Obesity/
14. exp Smoking/
15. exp Health Status/
16. Life Expectancy/ or Mortality/
17. exp Health Services Accessibility/ or exp Healthcare Disparities/
18. 7 or 8 or 9 or 10 or 11 or 12 or 13 or 14 or 15 or 16 or 17
19. 6 and 18
20. limit 19 to (english language and humans and yr="2010 - 2023")

**Article selection flowchart**

Articles identified from MEDLINE (n = 1619)

**Identification**

Not relevant (n = 365)

Non-US (n = 844)

Not interested in disability (n = 146)

No ableism measure (n = 123)

Ineligible primary aim (n = 98)

After duplicates removed

(n = 1617)

**Screening**

Articles included in analysis (n = 41)

**Included**

*Not relevant*: e.g., reviews, commentaries, articles on diagnostic test discrimination, randomized control trials (on physical therapy interventions), etc.

*Non-US*: articles that would have otherwise been eligible with the exception that they focused on a population outside of the United States

*Not interested in disability*: e.g., articles framed in terms of disability as an outcome of potential public health importance, but ultimately did not focus on a disabled population, etc.

*No ableism measure*: articles did not measure any form of ableism or generate an ableism-related theme according to our ableism levels

*Ineligible* *primary aim*: e.g., articles with outcomes not related to health (e.g., employment discrimination), related to recruitment of disabled people into research studies, etc.

**References**

1. Van Der Horst M, Vickerstaff S. Is part of ageism actually ableism? *Ageing and Society* (2022) 42:1979–1990. doi: 10.1017/S0144686X20001890

2. Ames SG, Delaney RK, Houtrow AJ, Delgado-Corcoran C, Alvey J, Watt MH, Murphy N. Perceived Disability-Based Discrimination in Health Care for Children With Medical Complexity. *Pediatrics* (2023) 152:e2022060975. doi: 10.1542/peds.2022-060975
